# Central Iliac Arteriovenous Anastomosis for Hypertension: Targeting Mechanical Aspects of the Circulation

**DOI:** 10.1007/s11906-015-0585-6

**Published:** 2015-07-31

**Authors:** Vikas Kapil, Paul A. Sobotka, Manish Saxena, Anthony Mathur, Charles Knight, Eamon Dolan, Alice Stanton, Melvin D. Lobo

**Affiliations:** Barts BP Centre of Excellence, Barts Heart Centre, St Bartholomew’s Hospital, W Smithfield, London, EC1A 7BE UK; William Harvey Research Institute, Barts NIHR Cardiovascular Biomedical Research Unit, Charterhouse Square, Queen Mary University London, London, EC1M 6BQ UK; Department of Internal Medicine, Division of Cardiovascular Diseases, The Ohio State University, Columbus, OH 43210 USA; ROX Medical, 150 Calle Iglesia # A, San Clemente, CA 92672 USA; Department of Cardiology, Barts Heart Centre, St Bartholomew’s Hospital, W Smithfield, London, EC1A 7BE UK; Department of Medicine for the Elderly, Connolly Hospital, Mill Road, Blanchardstown, Dublin 15 Dublin, Ireland; Molecular and Cellular Therapeutics, Royal College of Surgeons in Ireland Medical School, 123 Saint Stephen’s Green, Dublin 2 Dublin, Ireland

**Keywords:** Hypertension, Arterial stiffness, Arteriovenous anastomosis, Arteriovenous fistula, Interventional devices, Coupler

## Abstract

Raised blood pressure is the leading attributable risk factor for global morbidity and mortality. Real world data demonstrates that half of treated patients are at elevated cardiovascular risk because of inadequately controlled BP. In addition to pharmacotherapy, certain interventional strategies to reduce blood pressure and cardiovascular risk in hypertension can be considered according to international guidelines. One of the newer technologies entering this field is a proprietary arteriovenous coupler device that forms a fixed flow arteriovenous conduit in the central vasculature. In this review, we examine the development of and rationale for the creation of a central arteriovenous anastomosis in patients with hypertension and review the proposed mechanisms by which it may ameliorate hypertension. We critically review the clinical trial evidence base to date and postulate on future therapeutic directions.

## Introduction

Raised blood pressure (BP) remains the leading attributable risk factor for mortality worldwide [1], despite an enormous evidence base [2] for the myriad of licensed classes of anti-hypertensive medications. Only half of treated hypertensive patients achieve controlled BP to international guideline-based targets [3, 4]. Furthermore, in patients with (pharmacologically) resistant hypertension (RHTN, taking ≥3 anti-hypertensive medication classes, including a diuretic at maximally tolerated doses [5–7]) who remain at excessive cardiovascular (CV) risk [8], there is a paucity of high-quality, endpoint-driven evidence to suggest that addition of a fourth medication (or more) is likely to control BP and reduce excess morbidity and mortality. Thus, novel therapeutic strategies, including non-medication approaches, are of broad interest.

Although there are current advances in improved iterations of established anti-hypertensive medication classes and entirely new medication classes for both established and recently apparent pathophysiological mechanisms [9], additional poly-pharmacy may be a less attractive approach in patients with RHTN. It is estimated that adherence to anti-hypertensive medications for primary prevention is only ~50 % after 2 years [10]. Dislike of life-long pharmacotherapy and adverse medication effects are some of the reasons thought to underlie this non-adherence [11], leaving such patients at elevated cardiovascular risk. The past decade has seen the introduction of a suite of interventional approaches for the management of hypertension that are at different stages of clinical development [12•]. Whilst the majority of these technologies aim to reduce BP via targeting of the autonomic nervous system [12•], a new device strategy targeting mechanical properties of the vascular system has demonstrated significant, sustained and safe blood pressure lowering in a randomized, controlled trial without sham treatment. This device addresses the mechanical loss of arterial compliance, representing a novel treatment strategy and presenting potential unique clinical value.

## Design, Rationale and Development of the Arteriovenous Anastomotic Coupler

The CE-marked ROX Medical arteriovenous (AV) coupler is a stent-like device made of nickel/titanium alloy (nitinol) that exhibits shape memory to self-expand on deployment into a pre-formed configuration. It is deployed via percutaneous trans-arterial and -venous access from the external iliac vein into the adjacent external iliac artery, just above the level of the femoral head, to form a fixed diameter AV anastomosis delivering an arterial–venous shunt estimated to be ~0.8–1 L/min when sized to 4 mm in diameter [13]. The coupler remains constant in shape and the pressure gradient preserves constant flow. It was originally developed for the treatment of advanced chronic obstructive pulmonary disease (COPD) [14••, 15], with the aim of forming a fixed calibre, central AV anastomosis, conceived to increase central venous oxygenation. In so doing, arterial blood saturation is raised despite the venous blood flowing through pulmonary *shunts* that do not participate in gaseous exchange due to the parenchymal destruction in advanced COPD. Furthermore, the increase in venous return would cause a commensurate increase in cardiac output, therefore further increasing oxygen delivery to tissues [14••].

Initial pilot studies were conducted in two cohorts of patients with advanced/end-stage COPD. Whilst these initial studies demonstrated improvements in arterial blood oxygen content and tissue oxygen delivery, there were heterogeneous results with respect to functional improvements in exercise capacity [16, 15]. Although reduction in systemic vascular resistance [16] and increased cardiac output were demonstrated [16, 15], the effects on systemic arterial pressure were not reported. These initial proof-of-concept studies were performed using either end-to-side surgical AV anastomosis and/or a coupler sized to 5 mm in diameter (i.e., larger than the current iteration) with an earlier method of procedural implantation in advanced/end-stage COPD [16, 15]. These patients have different clinical risks/benefits and certainly their physiology is appreciably different to patients with RHTN. Whilst the procedural adverse event rates in these pilot COPD trials are notable (and includes ipsilateral lower limb oedema (67 %), venous stenosis (47 %), right heart failure (27 %) and deep venous thrombosis (27 %)), they cannot simply be translated to other patient groups with current device/delivery modifications.

Historical reports show that experimental very large-calibre, non-fixed diameter AV anastomoses in canines caused significant, sustained reduction in BP, though the maladaptive responses to such fistulae were not desirable [17, 18]. These included an increase in cardiac output and heart rate as well as an increase in total blood volume. In the case of larger or more proximal fistulae, the resultant hypotension could even be fatal or eventually cardiac dilatation and high output cardiac failure would ensue. In modern medical practice, the most common reason for formation of an iatrogenic AV anastomosis is to provide vascular access (AV fistula or AVF) in patients with end-stage renal disease (ESRD) for the purpose of haemodialysis (HD). Closure of AVFs in stable HD patients post-renal transplantation is associated with elevation in BP [19] and formation and maturation of upper limb AVFs in pre-dialysis patients is associated with significant peripheral BP reduction [20••, 21] and, in addition, reduction in central BP and aortic pulse wave velocity [20••], both validated measures of the effects of arterial stiffness [22]. High volume, upper limb AVF formation for HD has been associated with enlarged myocardial dimensions and reduction in endothelial function [23]. However, upper limb AVFs for HD are non-fixed calibre and dilate over time, have pressure-variable diameters and blood flow resulting in increased shunt fractions as blood pressure rises. When fistula flow as a proportion of cardiac output exceeds 30 %, screening for high output failure is often conducted; however, cardiac failure is seldom reported unless the shunt volume is greater than 2 L/min [24]. The ROX coupler delivers a fixed diameter conduit that can be upsized only through further balloon dilatation but will not expand spontaneously, resulting in anastomosis flow rates [13] that are unlikely to result in cardiac decompensation,

## Evidence for Blood Pressure Reduction in Hypertension

Whilst neither of the two initial published studies in normotensive COPD patients reported changes in systemic arterial BP, an interesting signal was apparent in a pooled cohort from two separate clinical trials in COPD patients (clinicialtrials.gov NCT00832611 and NCT00992680; total *n* = 95). In post hoc analyses of 24 patients with co-existent (systemic arterial) hypertension (mean resting BP 145/86 mmHg), insertion of the AV coupler (sized to 4 mm) was associated with 13/18 mmHg reduction in resting BP at 12 months post-central AV anastomosis formation [25]. More importantly, this effect was consistent with 67 % patients having >10 mmHg systolic blood pressure (SBP) reduction and 83 % patients having >10 mmHg diastolic blood pressure reduction, and negligible BP reduction in normotensive patients [26, 25]. The adverse event rate was lower than in the previous studies, with 17 % developing venous stenosis proximal to the stent insertion point that required either balloon venoplasty and/or stenting and 17 % developing deep vein thrombosis [25].

Following these observations, a small, prospective study (unpublished) in eight RHTN patients (mean 4 anti-hypertensive medications) was conducted using both office and ambulatory BP measurements to 6 months post-AV anastomosis (4 mm diameter) formation. Office BP (OBP) and ambulatory BP (ABP) decreased by 17/13 mmHg and by 6/13 mmHg, respectively [27], with some improvements in echocardiographic measures of diastolic function and left ventricular hypertrophy in a small subset (*n* = 5) [27].

These pilot data spurred the design of a larger, international, multi-centre, open-label, randomized, controlled trial in patients with confirmed office and out-of-office RHTN. In the ROX CONTROL HTN trial [28••], 83 patients were randomized in a 1:1 fashion to receive standard care (medication continuation) or insertion of the AV coupler plus standard care until the co-primary endpoint analysis (OBP and ABP) at 6 months, and a modified intention to treat analysis was performed in the 77 patients with complete data at this time-point.

The trial cohort was well balanced between the limbs and the patients had significant hypertension despite taking >4.6 medications. Baseline OBP was 175/100 mmHg, with out-of-office RHTN confirmed by ABP (24 h mean 157/93 mmHg). OBP was significantly reduced by 27/20 mmHg and ABP by 14/14 mmHg from baseline in the active treatment group, with no significant change in the control group [28••]. The magnitude of BP reduction observed at 6 months post-intervention was similar to those achieved through first-generation renal sympathetic denervation (RSD) technology and second-generation baroreflex activation therapy (BAT) [29, 30]. In a subset of patients (*n* = 10) who had previously received RSD, there was an equivalent BP response [28••], suggesting that inadequate response to one interventional technology does not preclude response to another, as had previously been demonstrated with BAT following inadequate response to RSD [29]. Importantly, the similar reduction in both SBP and DBP following central AV anastomosis formation preserves pulse pressure (PP), and therefore, long-term evaluation of these effects on target organ damage, arterial stiffness and CV events is required to demonstrate that maintenance of large PP (with lower mean arterial pressure) is beneficial overall. An initial case report of the effects of central AV anastomosis formation on parameters of arterial stiffness 4 months post-AV coupler insertion suggest that reduction in pulse wave velocity was independent of associated BP-lowering [31]. In the control group, five admissions for hypertension crisis occurred within the primary analysis 6-month period, whilst none occurred in the intervention group. Furthermore, there was medication reduction at 6 months in 25.6 % (*p* = 0.03) of patients in the intervention group whilst in the control group, 29.4 % (*p* = 0.04) of patients increased antihypertensives. This may have led to an underestimate of the true BP-lowering effect of the coupler as BP analysis was done independent of medication changes [28••].

Procedural complication rates were common (31 %) and included an ipsilateral iliac artery dissection, which required no further intervention (*n* = 1), severe contrast reaction in a patient with known dye allergy (*n* = 1) and localized time-limited pain (*n* = 2). Late, device-related events were exclusively related to venous stenosis causing limb oedema in 29 % of patients that were treated successfully by balloon venoplasty and/or stenting. Venous stenosis is caused by venous neointimal hyperplasia (VIH) and is a leading cause of AVF dysfunction in ESRD, with 2-year patency rates of only 75 % [32], and is a leading cause of both morbidity and hospitalization in ESRD patients [33]. Another potential mechanism for ipsilateral limb oedema is venous hypertension from impaired venous return distal to the anastomosis. Compression stockings are advised in all patients after device insertion, and some suggest co-administration of aspirin [25] or diuretics (current Barts BP Centre practice) to all patients to prevent these issues, though the efficacy of these approaches are as yet unknown.

ROX CONTROL HTN did not include a sham-control as the most recent Food and Drug Administration (FDA) Investigational Device Exemption (IDE) trial for radio-frequency (RF) RSD was designed to do. However, in the case of the AV coupler technology, a sham-controlled study may not be necessary or feasible. Firstly, upon opening of the anastomosis, there is immediate imaging confirmation of anatomical success of the procedure and this is confirmed by immediate, on-table, large reduction in BP [13], unlike RSD where technical success remains unverified at the time of the procedure. This immediate blood pressure finding restricts possible influences of the Hawthorne or placebo effect. Secondly, this therapy leads to a palpable thrill in the ipsilateral groin that can be felt by the patient [13].

## Proposed Mechanism of Beneficial Haemodynamic Effects in Hypertension

The mechanisms that regulate BP and can contribute to the pathophysiology of hypertension and RHTN are complex and manifold [34]. Two themes that have gained particular attention over the past two decades are the roles of the autonomic nervous system and large vessel arterial stiffening in the initiation, maintenance and resistance to pharmacotherapy of hypertension phenotypes.

Tonic elevation of sympathetic tone is widely recognized as a hallmark of hypertension, particularly in renal- and obesity-driven forms and in the young [35]. Furthermore, there is a clear association of increased indirect measures of sympathetic overactivity in uncontrolled (pharmacologically) RHTN [36, 37]. These abnormalities drove the development of two contrasting sympathomodulatory approaches for RHTN: RSD [38] and BAT [39]. Whilst these technologies have accrued substantial evidence of BP reduction in most studies to date and recommendations for use are included in recent international guidelines [6], sympathomodulation using RSD has been shown significantly less effective in terms of BP reduction in the context of isolated systolic hypertension (ISH) [40, 41], a common finding in middle-aged and older patients with hypertension [42]. There are multiple mechanisms that may be responsible for BP modulation after creation of a central AV anastomosis [43••]. Whilst it is predominantly thought that the BP reduction is related to mechano-circulatory improvements [28••], there is the possibility that neurally mediated vasomotor and hormonal alterations also play a role.

### Importance of Arterial Stiffness to Age-Related BP Elevation

**Fig. 1:**
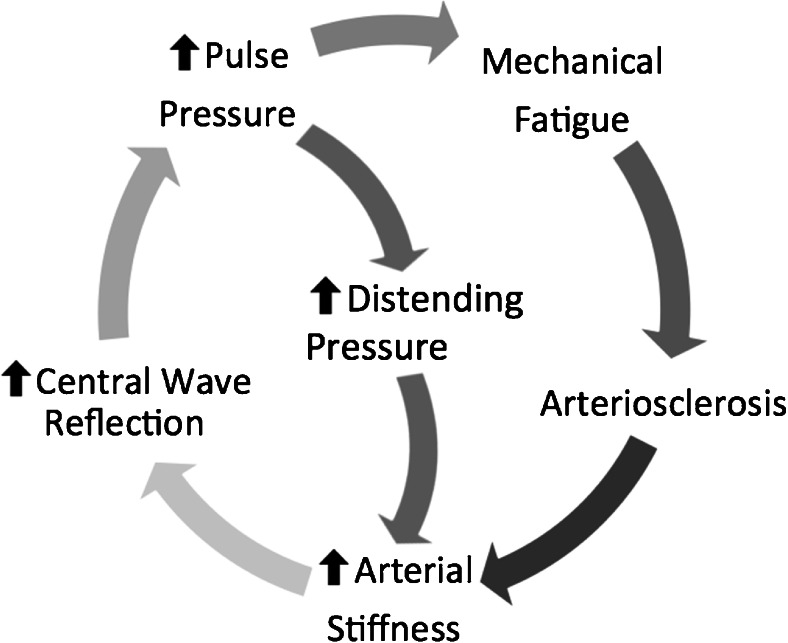
Feed-forward loop involving blood pressure parameters, circulatory damage and arterial stiffness

Large, central artery stiffening is due to elastin fatigue, collagen deposition and medial calcific arteriosclerosis [44, 45], and it precedes incident hypertension in large, prospective cohorts [46, 47]. Recent computational studies suggest that large artery stiffening alone is sufficient to explain both age-related increases in BP and failure of normal regulatory mechanisms (primarily renal and baroreflex mediated) to provide homeostasis [48]. Furthermore, arterial stiffness is implicated as a leading determinant of resistance to conventional pharmacotherapy, as most anti-hypertensive medications work at the level of the muscular arterioles rather than the central, elastic, large arteries [49••]. Central aortic elastic disorders inherently raise blood pressure, pulse pressure, pulse wave velocity all contributing to increases in volume sensitivity [50] and blood pressure variability [51] (Fig. 1). Loss of aortic elasticity is a component of ageing and may well explain the age-related increase in pulse pressure and loss of responsiveness to neurohumoral interventions.

**Fig. 2:**
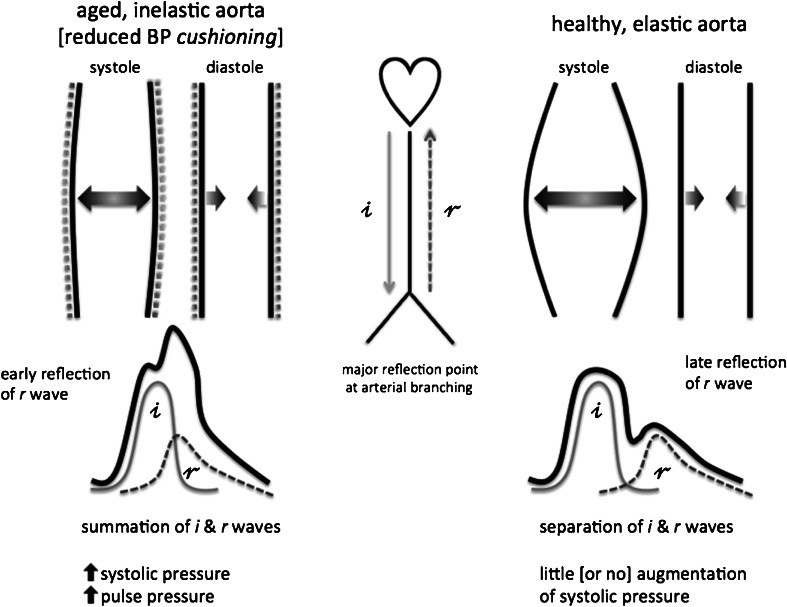
Modifying effects of arterial ageing/stiffness on the arterial pressure waveform. Incident (*i*) pressure waves leave the heart during systole and are accommodated by the Windkessel effect in young elastic arteries. Reflected (*r*) waves from arterial branching points arrive late in relation to the *i* wave and do not contribute to pressure amplification. In aged, stiff arteries, there is a loss of BP cushioning and early reflection of *r* waves, leading to increased systolic and pulse pressures (*BP* blood pressure)

Impedance mismatch in the distal aorta, predominantly at major arterial branching points, leads to pressure-wave reflection and return of a backward wave that augments systolic BP on top of the forward, propagation wave that leaves the heart [52]. This augmentation of central pressures is further increased in the setting of central arterial stiffness, as the lack of arterial compliance accelerates the velocity of both the forward and backward pressure waves, leading to more overlap of the distinct pressure waves in systole, leading to greater PP amplification and thus giving rise to the ISH phenotype [52] (Fig. 2). Furthermore, there is a contribution to central arterial stiffness arising from the distending pressure of the pressurized, blood-filled aorta [53], and thus, there is a potential for a negative feed-forward loop in this regard (Fig. 1).

Loss of arterial compliance necessarily involves loss of the *Windkessel* effect within the central arterial vasculature [54]. This loss of pressure dampening (*BP cushioning*) increases rate of pressure change within systole in the aorta and predisposes to increased systolic BP levels, greater BP variability and enhanced volume sensitivity of BP levels [55] (Fig. 2). The resultant magnification of peak systolic BP, PP and BP variability predisposes to organ damage, such as left ventricular hypertrophy, and increased risk of CV events [56].

### Central AV Anastomosis: Targeting Mechano-circulatory Aspects

There are several mechanisms by which creation of a central, fixed calibre AV anastomosis will target abnormal mechano-circulatory aspects of vascular physiology in hypertension that are likely to be additive and work in a beneficial feed-forward loops.

The addition of a low-resistance, high-compliance venous segment in parallel to the systemic arterial circulation will reduce overall systemic vascular resistance (SVR) (as has been demonstrated with the formation of a central AV anastomosis in the original pilot studies [16]), similar to Ohm’s law of electrical resistance. With a more proximal anastomosis, the reduction in SVR will be relatively greater than for distal circulatory AV conduits (such as upper limb AVFs) in relation to the contribution of vessel length to flow through a fixed diameter vessel/tube (Poiseuille’s Law). With increased venous return, there is activation of the Starling mechanism to increase cardiac output [16, 15, 20••, 21], equivalent to the anastomosis flow rate (~1 L/min); however, if the increase in cardiac output is not commensurate with the reduction in SVR caused by the addition of the parallel, low-resistance circuit, then mean arterial pressure will drop as is the case with HD AVF formation as previously discussed [20••]. Cardiac afterload is thereby reduced through a variety of mechanisms, including reduction of effective arterial volume and arterial stress, slowing of reflected pressure waves and preventing their systolic stacking. Importantly, reduction in effective arterial volume achieved through a central AV anastomosis does not come at the expense of reduction in whole-body volume affecting the intracellular, interstitial and venous capacitance compartments as typified by use of diuretics which are known to result in a concomitant increase in sympathetic outflow and may be responsible for attenuation of anti-hypertensive effect [57, 58].

**Fig. 3:**
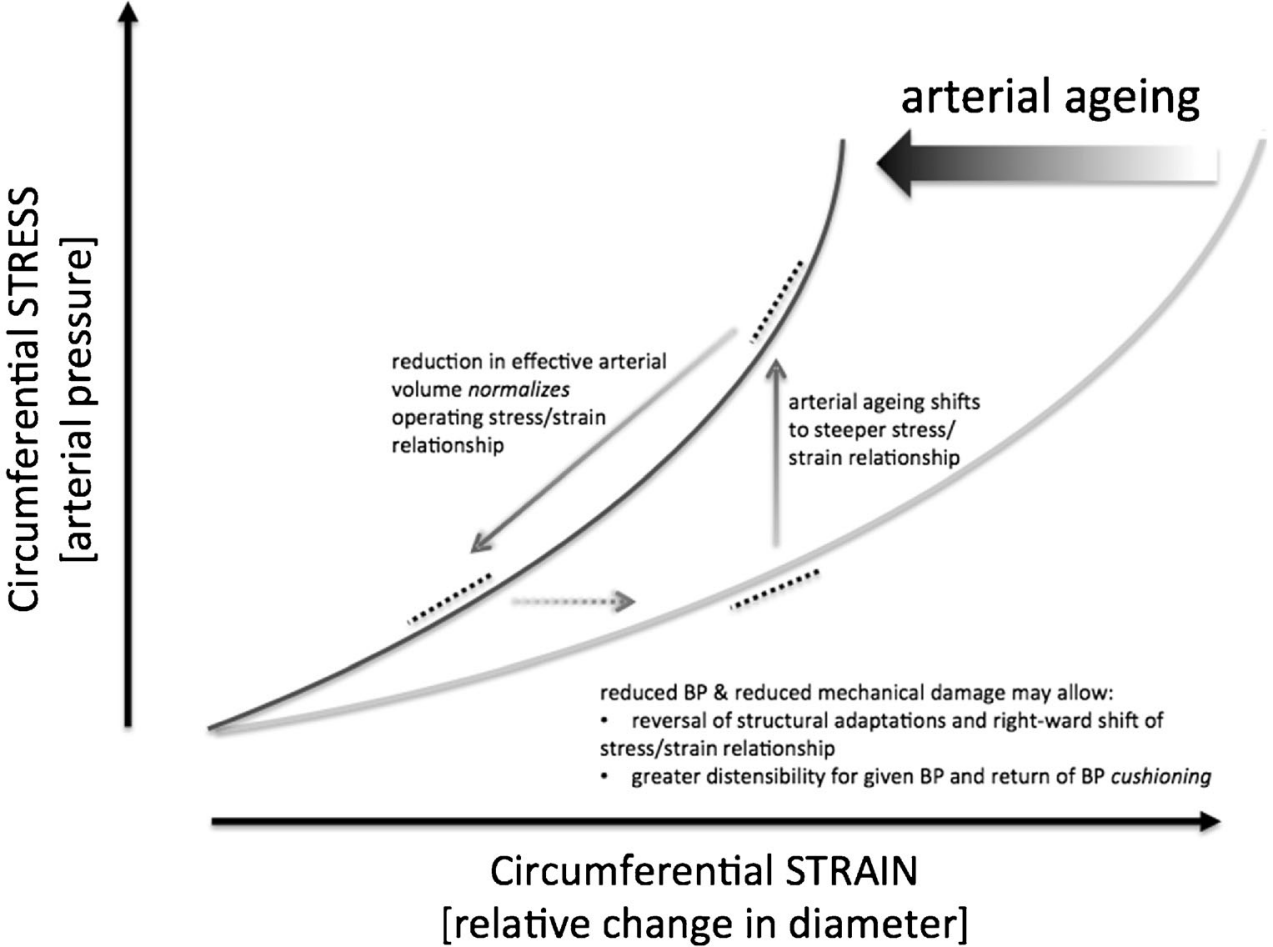
Age-related changes in the stress–strain relationship may be modified by central arteriovenous anastomosis. Age-related arterial stiffness moves the stress–strain relationship in the major elastic arteries to the left, reducing distensibility for a given BP. Effective reduction in arterial volume shifts the operating curve of the stress–strain relationship downwards, resulting in a pseudonormalisation of relationship. Further arterial remodeling may allow the stress–strain relationship to drift back towards the right towards normality allowing greater compliance for a given BP (*BP* blood pressure)

Perhaps most importantly, merely reducing effective arterial volume and cardiac afterload with a central AV anastomosis will alter the strain–stress intercept that reflects the non-linear volume/pressure dependency that determines arterial compliance (the reciprocal of arterial stiffness). This relationship is pathophysiologically shifted leftwards in hypertension related to arterial stiffening (Fig. 3). This change will allow for some improvement in BP cushioning and return of a Windkessel-like effect, further reducing pulse pressure amplification due to a delayed return of the reflected wave and reduced pressure stacking. Reduction of distending BP in the aorta will also further improve arterial compliance, and therefore, there is a possibility of a feed-forward loop occurring between reduced SVR, improved compliance, and reduced distending BP and the long-term possibilities of beneficial aortic remodeling (Fig. 3).

### Central AV Anastomosis: Neurohumoral and Sympathomodulatory Aspects

In addition to several mechano-circulatory aspects that may contribute to mean BP and BP variability improvements discussed above, there may well be anticipated benefits from neurohumoral and sympathomodulatory aspects. Increased flow to the cardio-pulmonary circuit will stimulate release of atrial natriuretic peptide, which may augment BP reduction as it is a potent vasorelaxant peptide that additionally has tubular effects in the glomerulus to reduce sodium reabsorption [59]. Furthermore, increased cardiac output resulting in stimulation of right atrial baroreceptors (via the Bainbridge reflex) and possibly cardiac vagal mechanoreceptors (via increased parasympathetic tone) may cause a beneficial sympathomodulatory effect on natriuresis and vasomotor tone [60].

The combination of increased cardiac output [13] and arterial blood oxygen content [16] will improve delivery of oxygen to various tissue beds, with possible beneficial effects, mediated in part by inhibition of peripheral carotid chemoreceptor activity, which augments sympathoexcitation and is reduced with hyperoxia [61]. This arises by improving renal oxygenation and thus downregulating afferent sympathetic signalling from ischaemia-sensitive renal chemoreceptors [62] and by reducing hypoxia-dependent pulmonary arterial vasoconstriction that would facilitate further improvements in systemic oxygenation.

## Future Directions

In common with similar early-phase investigations of interventional strategies for hypertension, ROX CONTROL HTN was designed to evaluate safety and efficacy of a primary endpoint and not designed to evaluate reductions in target organ damage or hard CV endpoints, and therefore, longer-term follow-up in global registries and investigator-led studies will be necessary to demonstrate an equivalent evidence base to guideline-based pharmacotherapy [2]. In addition, reporting on late complications is pending further follow-up on the reported cohort. A FDA, US-based IDE trial is in development and will help better define the optimal target population(s) and expected benefit and risks. A global registry (RH03) has started recruitment with the aim of entering 100 RHTN patients with ongoing follow-up to determine long-term efficacy and safety (clinicaltrials.gov: NCT01885390) and may allow for the inclusion of other hypertension phenotypes, such as multi-drug intolerant patients, for whom there are extremely limited treatment options [63]. Additional, serial testing will be performed at some sites to include detailed cardiac structural evaluation, to determine whether non-fixed calibre AVF formation in non-ESRD patients is associated with previously observed maladaptive changes [23]. Non-invasive autonomic testing with simultaneous beat-by-beat haemodynamic assessment could in addition determine whether the observed BP-lowering effects are purely mechano-circulatory or involve sympathomodulation as well.

Other avenues that are being explored in nascent clinical trials include evaluating the effects of the coupler in paroxysmal atrial fibrillation (AF), as an adjunct to AF ablation (clinicaltrials.gov: NCT02243891) and in neurally mediated syncope (clinicaltrials.gov: NCT02388087) where it is postulated that increases in cardiac pre-load will reduce symptoms. Due to the expected reduction in cardiac afterload related to reduction in SVR and improvements in arterial compliance, it has been suggested that improvements in cardiac mechanics and reductions in myocardial oxygen consumption may be apparent [13], which would provide the possibility of an additional research stream in refractory angina, though to date, no pre-clinical or clinical data has been presented to demonstrate these effects.

In common with more established interventional technologies in the field of hypertension, such as RSD and BAT, iteration of the device and deployment technique may be possible to improve efficacy and enhance safety. One possibility is for graded sizing of the AV anastomosis, so that patients first have a smaller calibre anastomosis formed (e.g., 2–3 mm diameter) and that if BP reduction related to this effect is not sufficient to control BP to guideline-driven targets, then the anastomosis could be further dilated to achieve the necessary haemodynamic effect. Furthermore, procedural modification is desirable to minimize venous trauma and modulation of artery-to-vein flow to avoid neo-intimal hyperplasia in order to reduce the prevalence of venous stenosis.

## Conclusions

Formation of a central AV anastomosis via a proprietary anastomotic coupler device has joined the burgeoning suite of interventional strategies for the reduction of BP in (pharmacologically) RHTN. Although its beneficial effects are likely to be related to mechano-circulatory improvement in SVR and arterial compliance, there is the possibility that it is sympathomodulatory in addition. Clinical trial data to date is encouraging, but both regulatory authorities and international guideline-writing societies will require further safety and hard CV end-point data before it becomes an established weapon in the anti-hypertensive armamentarium.
